# Magnitude and factors associated with sexual re-victimization among adolescent girls and young women in Kinshasa, Democratic Republic of the Congo: a retrospective multicenter study

**DOI:** 10.1186/s12978-023-01710-z

**Published:** 2023-12-06

**Authors:** Fifiya Biluala, Harry César Kayembe, Doudou Batumbo, Germain Kapour, Félicitée Mumbanza, Eric Bokabo, Benjamin Longo-Mbenza, Berthe Zinga

**Affiliations:** 1 Master of Ecology and Governance of Infectious Diseases, Faculty of Medicine, University of Kinshasa, Kinshasa, Democratic Republic of the Congo; 2 Department of Basic Sciences, Faculty of Medicine, University of Kinshasa, Kin XI, B.P.: 834, Kinshasa, Democratic Republic of the Congo; 3 Department of Internal Medicine, Faculty of Medicine, University of Kinshasa, Kinshasa, Democratic Republic of the Congo; 4 Department of Gynecology and Obstetrics, Faculty of Medicine, University of Kinshasa, Kinshasa, Democratic Republic of the Congo

**Keywords:** Sexual re-victimization, Adolescent girls, Young women, Magnitude, Associated factors, Democratic Republic of the Congo

## Abstract

**Background:**

Adolescent girls and young women are more exposed to sexual violence. A significant proportion of victims of sexual abuse are victims of sexual re-victimization. However, information on the burden of sexual re-victimization among AGYW in contexts other than conflict-affected areas in the Democratic Republic of the Congo (DRC) is limited. The aim of this study was to assess the magnitude of sexual re-victimization among AGYW and to identify associated risk factors in the capital, Kinshasa.

**Methods:**

We conducted a retrospective multicenter cohort study in which sexual violence records between 2015 and 2020 were used to extract and analyze victims’ sociodemographic and behavioral characteristics and profiles of sexual violence perpetrated. Multivariate logistic regression models were employed to identify factors associated with sexual re-victimization using the adjusted odds ratio (AOR) with its 95% confidence interval (95% CI) and *p* value < 0.05.

**Results:**

We found that 74 (31%) of the 241 AGYW included in this study had experienced sexual re-victimization. Sexual re-victimization was associated with being older (> 19 years), sexually active, and living in a single-parent family, and with perpetrator types, particularly intimate partners and family members.

**Conclusions:**

Our findings provide tools for developing and implementing targeted prevention and intervention programs to reduce sexual violence in general and sexual re-victimization in particular.

**Supplementary Information:**

The online version contains supplementary material available at 10.1186/s12978-023-01710-z.

## Background

Sexual violence remains a major global public health concern and a widespread violation of human rights. It refers to any forced sexual act, including attempted or completed rape, coercion and harassment, as well as other forms of sexual contact with force or threat of force by any person, regardless of their relationship to the victim, in any context [1]. Sexual violence damages the physical and psychological integrity of victims, and has harmful consequences such as post-traumatic stress disorder (PTSD), depression, anxiety, suicidal thoughts, self-harm, poor school or academic performance and substance abuse [2–5].

Research shows that females are at higher risk for sexual victimization, particularly in adolescence and youth [6–9]. Globally, during the period 2000–2018, more than 641 million women aged 15 and over, including nearly one in four adolescent girls, reported having been victims of sexual violence [10]. The highest prevalence rates of sexual violence against adolescent girls and young women (AGYW) are observed in a few regions, particularly in Sub-Saharan Africa (SSA). Several studies carried out in different countries of SSA report prevalence rates ranging from 25% to over 60% among AGYW [7, 11–15]. The Democratic Republic of the Congo (DRC), plagued by more than two decades of violence and recurrent socio-political crises, is one of the worst-affected countries [10].

It should be noted that previous victims of sexual violence are particularly vulnerable to sexual re-victimization [6, 16–18]. The risk of re-victimization is three to five times greater among AGYW who have reported sexual abuse histories [6, 16, 19, 20]. However, most of the existing literature, with a particular focus on Congolese AGYW, describes the extent of sexual violence in conflict-affected areas [21–23]. Sexual violence, let alone repeated sexual violence, among AGYW would therefore be under-reported and under-investigated in other contexts. As sexual re-victimization is associated with an exacerbation of subsequent physical and psychological abuse, it is crucial to study their risk factors in order to improve prevention programs and, consequently, psychosocial well-being. Accordingly, the aim of the present study was to assess the magnitude of the burden of sexual re-victimization among AGYW who have experienced sexual violence and to identify their associated factors in the city of Kinshasa, the capital of the DRC.

## Methods

### Study design, setting and period

**Fig. 1:**
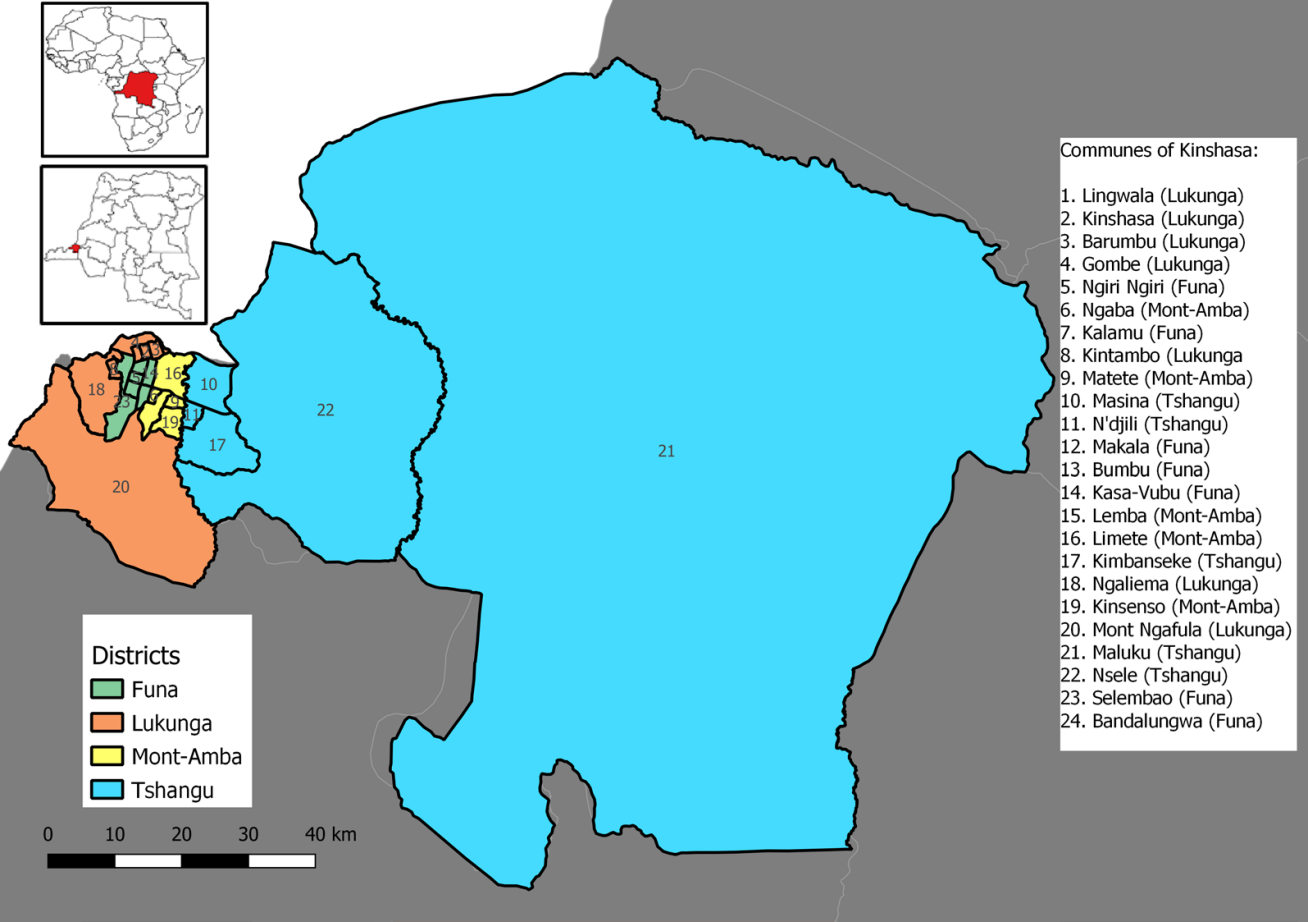
Administrative map of the study area

 A retrospective multicenter cohort study was conducted in governmental and non-governmental institutions dealing with gender-based violence, specifically medical, psychosocial, legal/judicial, and social reintegration facilities for victims of sexual violence located in the four districts of Kinshasa (Fig. 1; Table 1). The study covered the period from January 1, 2015 to December 31, 2020.

**Table 1:** Districts, institutions and facilities for dealing with sexual violence, and types of support

Districts	Institutions and facilities	Types of support
Funa	Association pour le Bien-Etre Familial/Naissances Désirables (ABEF-ND)	Medical and psychosocial
Lukunga	Hôpital Général de Référence de Kintambo	Holistic
	Ligue de la Zone Afrique pour la défense des Droits des Enfants et Elèves (LIZADEEL)	Legal/judicial and psychosocial
	Police de Protection d’Enfant et de Prévention des Violences Sexuelles (PEPVS)	Legal/judicial and psychosocial
Mont-Amba	Hôpital Saint Joseph	Medical and psychosocial
	Centre Mère et Enfant de Ngaba	Holistic
	Centre Féminin Marie Antoinette	Medical and psychosocial
	UMOJA	Social reintegration
Tshangu	Hôpital Général de Référence de Ndjili	Holistic

### Study sample, participants and inclusion criteria

The study sample was obtained after analysis of intake records, counseling forms, court and medical records, and follow-up documentation of consecutive (serial) cases of sexual abuse among AGWY. The latter consisted in detecting the consequences of sexual violence on the victim’s physical and mental health, either within 72 h of the incident or beyond, in order to ensure appropriate medical, psychological and social care.

The study involved 300 females aged between 10 and 24 who had been victims of sexual violence and were being cared for in governmental and non-governmental facilities dealing with gender-based violence. Fifty-nine participants were excluded due to lack of usable information in the records or verbal informed consent to participate in the study, reducing the final sample size to 241.

### Data collection

An ad hoc questionnaire was elaborated to collect information on sociodemographic and behavioral characteristics of the victims and the profiles of the sexual violence perpetrated. The following variables were recorded:

- Sociodemographic and behavioral characteristics of victims: age, place of residence, educational level, marital status, family structure, sexual activity, and drug use.
- Profiles of sexual violence perpetrated: place of occurrence, perpetrator type, and experience of sexual re-victimization.

The collected information was finally entered into Microsoft Excel® 2019 files for data management before analysis.

### Operational definitions

- Sexual re-victimization: experience of subsequent sexual victimization throughout the lifespan following initial sexual violence [17, 18, 24].
- Place of residence: characterized as rural, peri-urban, and urban based on typology of communes in the urban-rural gradient according to their urbanization morphology [25]. The average population density in the areas represented by the communes of residence is low, intermediate and high, respectively [25].

### Statistical analyses

Statistical analyses for this study were carried using R® version 4.2.0. First, we used descriptive statistics to describe the sociodemographic and behavioral characteristics of the study participants, as well as the profile of sexual violence perpetrated in terms of frequency and percentage. Second, we performed binary logistic regression models, with bivariate and multivariate analyses, to assess the association between covariates and sexual re-victimization among AGYW. Crude odds ratios (COR) and adjusted odds ratios (AOR) with their 95% confidence intervals (95% CI) were determined and statistically significant associations were declared at *p* value < 0.05. The final multivariate logistic regression model was built with a reduced number of predictor variables in order to improve the explanatory model through automatic selection using a step-by-step top-down procedure based on minimization of the Akaike information criterion (AIC).

## Results

### Sociodemographic characteristics of AGYW included in this study

A total of 241 AGYW were included in this study. Their mean age was 15.3 (± 3.07) years. The majority of AGYW were aged between 15 and 19 (50.2%), lived in urban areas (61.8%), had completed formal secondary education (68.9%), and were single (95%). However, 21.6% and 12.0% of these AGYW were sexually active and using drugs, respectively (Table 2).

**Table 2:** Sociodemographic and behavioral characteristics of AGYW

Variables	Frequency	Percentage
Age groups		
10–14 years	98	40.7
15–19 years	121	50.2
20–24 years	22	9.1
Place of residence		
Rural	71	29.5
Peri-urban	21	8.7
Urban	149	61.8
Educational level		
No formal education	6	2.5
Primary completed	67	27.8
Secondary completed	166	68.9
Tertiary completed	2	0.8
Marital status		
Single	229	95.0
Married	12	5.0
Family structure		
Single-parent family	136	56.4
Two-parent family	54	22.4
Other	51	21.2
Sexually active		
No	189	78.4
Yes	52	21.6
Drug use		
No	212	88.0
Yes	29	12.0

## Characteristics of sexual violence and magnitude of sexual re-victimization among AGYW

Table 3 shows that more than half of all sexual assaults were committed in the abuser’s own home (52.7%). The majority of sexual assaults were perpetrated by an acquaintance (31.1%), followed by an intimate partner (21.6%) and a stranger (20.3%). In addition, 74 (31%) of the AGYW included in this study had experienced sexual re-victimization.

**Table 3:** Characteristics of sexual violence perpetrated

Variables	Frequency	Percentage
Place of occurrence		
Abuser’s home	127	52.7
Survivor’s home	14	5.8
Other	100	41.5
Perpetrator type		
Acquaintance	75	31.1
Family member	24	10.0
House neighbor	41	17.0
Intimate partner	52	21.6
Stranger	49	20.3
Experience of sexual re-victimization		
No	167	69.3
Yes	74	30.7

### Factors associated with sexual re-victimization among AGYW

Table 4 presents the results of bivariate logistic regression analysis on the association of victim characteristics and sexual violence profile with sexual re-victimization among AGYW in Kinshasa. It revealed that variables such as age, sexually active, and perpetrator type showed statistically significant associations with sexual re-victimization.

**Table 4:** Bivariate logistic regression analysis of the association of victim characteristics and profile of sexual violence with sexual re-victimization status among AGYW in Kinshasa, 2015–2020

Variables	Sexual re-victimization		COR (95% CI)	*p*
	No n (%)	Yes n (%)		
Age groups				
10–14 years	74 (75.5)	24 (24.5)	1	
15–19 years	83 (68.6)	38 (31.4)	1.41 (0.78–2.59)	0.260
20–24 years	10 (45.5)	12 (54.5)	3.70 (1.43–9.84)	**0.007**
Place of residence				
Rural	52 (73.2)	19 (26.8)	1	
Peri-urban	16 (76.2)	5 (23.8)	0.86 (0.25–2.53)	0.80
Urban	99 (66.4)	50 (33.6)	1.38 (0.75–2.63)	0.30
Educational level				
Primary completed and below	55 (75.3)	18 (24.7)	1	
Secondary completed and above	112 (66.7)	56 (33.3)	1.53 (0.83–2.90)	0.20
Sexually active				
No	151 (79.9)	38 (20.1)	1	
Yes	16 (30.8)	36 (69.2)	8.94 (4.57–18.20)	**< 0.001**
Marital status				
Single	158 (75.0)	71 (25.0)	1.35 (0.39–6.21)	0.70
Married	9 (69.0)	3 (31.0)	1	
Family structure				
Single-parent family	89 (65.4)	47 (34.6)	1.72 (0.84–3.71)	0.20
Two-parent family	39 (72.2)	15 (27.8)	1.25 (0.52–3.06)	0.60
Other	39 (76.5)	12 (23.5)	1	
Drug use				
No	148 (69.8)	64 (30.2)	1	
Yes	19 (65.5)	10 (34.5)	1.22 (0.52–2.71)	0.60
Place of occurrence				
Abuser’s home	86 (67.7)	41 (32.3)	2.86 (0.74–18.93)	0.20
Survivor’s home	12 (85.7)	2 (14.3)	2.70 (0.68–17.99)	0.20
Other	69 (69.0)	31 (31.0)	1	
Perpetrator type				
Acquaintance	52 (69.3)	23 (30.7)	2.27 (0.95–5.89)	0.076
House neighbor	30 (73.2)	11 (26.8)	1.88 (0.68–5.40)	0.228
Family member	12 (50.0)	12 (50.0)	5.13 (1.74–16.06)	**0.004**
Intimate partner	32 (61.5)	20 (38.5)	3.20 (1.29–8.61)	**0.015**
Stranger	41 (83.7)	8 (16.3)	1	

**Fig. 2:**
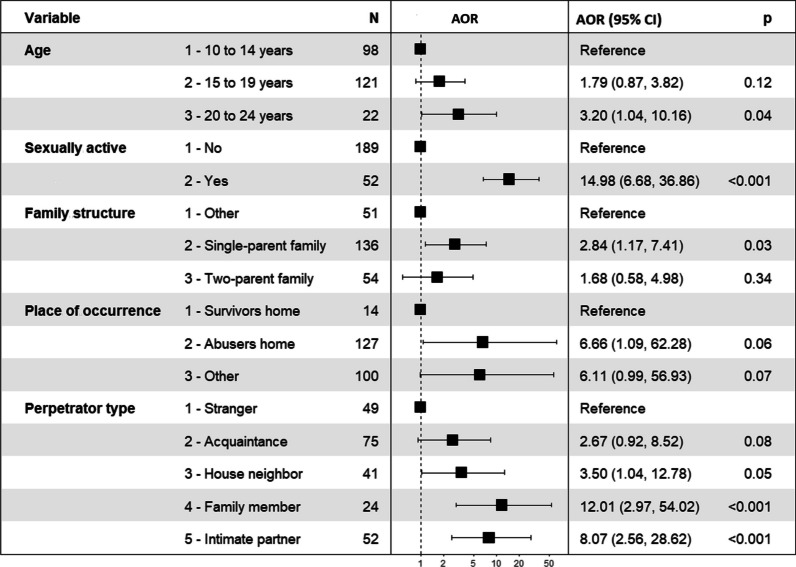
Factors associated with sexual re-victimization among AGYW identified by the final multivariate logistic regression model

 Figure 2 shows independent predictors of sexual re-victimization identified at the final multivariate logistic regression model. Sexual re-victimization among AGYW in Kinshasa was significantly associated with being aged 20 to 24 years (AOR = 3.20, 95% CI [1.04–10.16]), being sexually active (AOR = 14.98, 95% CI [6.68–36.86]), and living in a single-parent family (AOR = 2.84, 95% CI [1.17–7.41]). Moreover, AGYW were more likely to be exposed to sexual re-victimization perpetrated by a family member (AOR = 12.01, 95% CI [2.97–54.02]) and an intimate partner (AOR = 8.07, 95% CI [2.56–28.62]).

## Discussion

The present study showed that, of all AGYW who had been sexually abused, three out of ten had experienced sexual re-victimization. This finding is in line with the literature, which has shown an increased risk of re-victimization in AGYW with a history of sexual abuse [6, 16, 19, 20]. The relatively high magnitude of sexual re-victimization among AGYW observed in this study may be explained by the fact that sexual violence has a lasting negative impact on the victim’s mental health, in terms of perception of herself, of events, and others. Thus, adolescents and women who suffer from mental disorders precipitated and maintained by sexual violence continue to be exposed to further sexual violence [26].

This study highlighted that the risk of sexual re-victimization was higher in AGYW who were likely to be older (> 19 years) and sexually active. These results are consistent with other research suggesting that sexual abuse in childhood or adolescence increases the risk of future victimization in adulthood [6, 18, 27]. Moreover, women who experience an episode of childhood sexual abuse are engaged in a vicious circle that includes more self-blame, higher levels of PTSD, and riskier sexual behaviors. This potentiates the tendency to have a greater number of partners, increasing the risk of further episodes of sexual victimization in adolescence and adulthood. As the more partners they have, the more likely they are to be assaulted [28, 29].

We also found that AGYW who lived in a single-parent family were more likely to be exposed to sexual re-victimization. In the light of other studies suggesting plausible effects of family structure on exposure to sexual violence and abuse [30, 31], this finding could be related to the fact that AGYW living in single parent households may be exposed to a greater number of adult males than those living in two-parent households, putting them at greater risk of further sexual victimization.

In this study, AGYW were eight times more likely to be re-victimized by sexual violence perpetrated by an intimate partner than by a stranger. Our findings are consistent with other studies that have indicated that women who have experienced violence on one occasion in an intimate partner relationship are at higher risk of being assaulted again by the same partner and in further relationships [32]. This association may be explained by the fact that re-victimization in the form of an intimate partner violence (IPV) is mediated by PTSD in female survivors of sexual abuse [33, 34]. It should be noted that the experience of multiple episodes of IPV has more severe and longer-lasting psychological and emotional consequences than an occasional episode of violence [34–36].

Furthermore, AGYW were 12 times more likely to experience re-victimization of sexual violence perpetrated by a family member than by a stranger. The possible explanation for this finding could be that the frequent presence of a non-caregiving adult in the home, such as an extended family member, can facilitate the occurrence of childhood sexual abuse at an early age [17], which may increase vulnerability for further episodes of victimization in adolescence and youth [37].

### Strength and limitations of the study

To our knowledge, this study is the first to address the issue of sexual re-victimization among AGYW who have experienced initial sexual violence in non-conflict areas of a Sub-Saharan country plagued by recurrent socio-political violence such as the DRC. Interestingly, the fairly high frequency of sexual re-victimization experienced by AGYW found in the present study is almost similar to that reported in the literature. However, this study has some limitations. Firstly, the retrospective design and recruitment of a limited sample size mean that the results can only be generalized to AGYW who have been supported in governmental and non-governmental care facilities for victims of sexual violence. Further community surveys are needed for greater representativeness at subnational and national levels. Secondly, other factors of interest were not considered in this study either. Indeed, specific elements such as mental health outcomes [18], types of re-victimization [38], relationship types [39] have been suggested to play a role on trends and prediction of sexual re-victimization. There is need for future studies to evaluate them and include them (Additional file 1).

## Conclusion

The overall magnitude of sexual re-victimization among AGYW in Kinshasa was relatively high. Being over 19, sexually active, and living in a single-parent family were significantly associated with sexual re-victimization. Intimate partners and family members were the main perpetrators of sexual re-victimization among AGYW. Our findings provide a basis for policymakers to develop and implement evidence-based prevention and intervention programs that can further reduce sexual violence in general and revictimization in particular as major public health concerns to improve the overall well-being of AGYW. In addition, these findings can serve as a baseline for future community-based cross-sectional and longitudinal studies to determine the true burden of sexual re-victimization among AGYW.

### Supplementary Information

**Additional file 1.** Dataset S1.

## Data Availability

All data generated or analyzed during this study are included in this published article.

## Abbreviations

95% CI: 95% Confidence intervals
AGYW: Adolescent girls and young women
AOR: Adjusted odds ratios
COR: Crude odds ratios
DRC: Democratic Republic of the Congo
IPV: Intimate partner violence
PTSD: Posttraumatic stress disorder
SSA: Sub-Saharan Africa
